# A Comparison of the Effects of Estrogen and *Cimicifuga racemosa* on the Lacrimal Gland and Submandibular Gland in Ovariectomized Rats

**DOI:** 10.1371/journal.pone.0121470

**Published:** 2015-03-20

**Authors:** Yunmeng Da, Kaiyu Niu, Ke Wang, Guangxia Cui, Wenjuan Wang, Biao Jin, Yu Sun, Jing Jia, Lihua Qin, Wenpei Bai

**Affiliations:** 1 Department of Stomatology, General Hospital of Chinese People’s Armed Police Forces, Beijing, China; 2 Department of Anatomy and Embryology, Peking University Health Science Center, Beijing, China; 3 Department of Obstetrics and Gynecology, Civil Aviation General Hospital, Beijing, China; 4 Department of Obstetrics and Gynecology, Peking University First Hospital, Beijing, China; Oklahoma State University, UNITED STATES

## Abstract

This study aims to observe the effects of estradiol and *Cimicifuga racemosa* on the lacrimal gland and submandibular gland of ovariectomized rats. We randomly divided 20 adult female SD rats into four groups—a sham-operated group (SHAM), ovariectomized (OVX) group, ovariectomized group treated with estradiol (OVX+ E), and ovariectomized group treated with the isopropanolic extract of *Cimicifuga racemosa* (OVX+ iCR). The SHAM group and OVX group used distilled water to instead the drugs. Two weeks after ovariectomy, the estradiol and iCR were administered for 4 weeks. Next, we used H&E staining and electron microscopy to observe any histological changes in the lacrimal and submandibular glands and immunohistochemical staining to observe the expressions of cleaved caspase-3 (Casp-3) and Cu-Zn SOD (superoxide dismutase). The H&E staining find that both drugs can prevent the cells of area from shrinkage in the two kinds of gland. But under the electron microscopy, estradiol and iCR have different efficacy. Estradiol is more effective at protecting mitochondria in lacrimal gland acinar cells than iCR, and iCR is more effective at suppressing endoplasmic reticulum expansion than estradiol. Both estradiol and iCR have a similar protective function on mitochondria in the submandibular gland. The protective function of the two glands may inhibit apoptosis by suppressing the expression of Casp-3. In addition, iCR increases the expression of Cu-Zn SOD in duct system of submandibular gland. The results suggest that both estradiol and iCR confer a protective effect on the lacrimal and submandibular glands of ovariectomized rats via different mechanisms.

## Introduction

Menopause, which occurs at ages 45~55 years in women, is a physiological process defined by the cessation of menstruation for more than 6 months, caused by ovarian function diminishing naturally or after surgery [1]. In climacteric syndrome, in addition to the common symptoms such as hot flushes, night sweats, and dyspareunia, the symptoms of dry eyes and dry mouth also increase significantly. Previous studies have revealed that these discomforts were caused by postmenopausal hormone deficiency, which could induce a reduction in secretions of the lacrimal and salivary gland. Estradiol therapy can alleviate the symptoms of dry eyes and dry mouth in menopause. There was a marked increase in both the fundamental and stimulating tear secretion of menopausal women who had received hormone replacement therapy (HRT) for three months [2], and an increase in the labial gland saliva flow rate, total salivary gland secretory rate, and buffering capacity in menopausal women (aged 61–76 years) who had received HRT for one year [3]. These results clearly suggest that HRT can increase the secretion of tear and saliva.

However, the Women's Health Initiative has highlighted that long-term use of HRT may increase the risk of heart disease, stroke, thrombosis and breast tumors [4]. Recently, clinical data revealed that about 50% of menopausal women who were unwilling to accept HRT continuous treatment would accept other alternative therapies to relieve menopausal symptoms, particularly herbal preparations [5, 6, 7, 8]. *Cimicifuga racemosa*, also known as “black cohosh,” is a perennial herb that is native to North America. It is a member of the buttercup family (*Ranunculaceae*) and amongst the most thoroughly studied herbal treatment for menopausal symptoms. In Europe, its history for treating menopausal symptoms has been last for more than half a century [9, 10, 11]. Some research has shown that this herbal treatment has a similar curative effect to tibolone in reducing the severity of menopausal symptoms, and a lower incidence of adverse events than tibolone [12]. Moreover, *Cimicifuga racemosa* has been shown to have the same effect as estradiol valerate in the treatment of menopausal osteoporosis, without exhibiting the side-effects of estradiol valerate on the uterus and breast [13, 4]. Furthermore, it has been reported that *Cimicifuga racemosa* methanol extracts act as antioxidants to protect cells effectively against DNA damage caused by reactive oxygen species (ROS) and scavenged 1, 1- diphenyl-2-picrylhydrazyl free radical (DPPH) [14]. Nine antioxidant active compounds have been extracted from *Cimicifuga racemosa*, and could limit DNA damage mediated by menadione in S30 breast cancer cells in vitro, including methyl caffeate, caffeic acid, ferulic acid, cimiracemate A and cimiracemate B, e ct [14]. In addition, two triterpenoid glycosides (cimiside E, 23-O-acetylshengmanol- 3- xyloside) and one furanocoumarin (isoimperatorin) extracted from *Cimicifuga racemosa* can selectively inhibit the expression of VCAM-1 (vascular cell adhesion molecule 1) mediated by TNF-α(Tumor Necrosis Factor-α) in human epithelial cells, and inhibit the phosphorylation of ERK1/2 (extracellular signal-regulated kinases 1 and 2), PI3K (phosphatidylinositol 3-kinase), and PKC (protein kinase C) signal molecules by up-regulation of PPAR-γ (peroxisome proliferator-activated receptor gamma) [15]. These signaling pathways act in the same way as the estrogen pathways, which suggest that the similar effects of both *Cimicifuga racemosa* and estrogen may be associated with this. Nevertheless, little is known about the effect of *Cimicifuga racemosa* on the lacrimal and salivary glands. Due to their vulnerable characteristics, these glands are prone to be affected by a variety of drugs that lead to a decline in their function. In addition, *Cimicifuga racemosa* has a complex composition, and its mechanism has not yet been clearly understood. Therefore, an investigation into how the drug impacts on the lacrimal gland and salivary gland will help us to understand the mechanisms of *Cimicifuga racemosa* and guide its clinical application.

It is well known that increased oxidative stress (OS) is a key event in postmenopausal women. Previous research has found that the protective function of the female sex hormone on the central nervous system, and against coronary artery and heart diseases, is associated with its antioxidant capacity [16, 17, 18]. It was found that, in the parotid of female rats, estrogen could influence the production of Cu-Zn SOD (superoxide dismutase) [19]. *Cimicifuga racemosa* shares this antioxidant capacity. Therefore, we hypothesized that the higher incidence of dry mouth and dry eyes in menopausal women was related to OS, and also that treatment with estrogen and *Cimicifuga racemosa* could relieve such symptoms.

## Materials and Methods

### 1: Ethics Statement

The experimental protocols and procedures were approved by the Biomedical Ethics Committee of Peking University (the approval number: LA2012–82). The surgery and euthanasia were performed under 1% sodium pentobarbital intra-peritoneal injection anesthesia to minimize suffering. In the end of this experiment, all the rats were decapitated to death after the lacrimal and submandibular glands were excised.

### 2: Animal models

20 healthy adult female Sprague Dawley rats (purchased from the Department of Laboratory Animal Science of Peking University Health Science Center), aged 8–10 weeks and weighed 210–230g, were used in this study. Just as previously described, the rats were placed in the laboratory with the temperature of 25±1°C, the light/dark cycle of 12 h, and relative humidity of 40–50%. Besides, the rats were exposed to direct light and given free access to soy-free forage and water for 2 weeks [20].

### 3: Reagents and instruments

Rabbit anti-rat Cu-Zn SOD (SOD-1) (#SC-11407) polyclonal antibody was purchased from Santa Cruz (USA). The Rabbit anti-rat Casp-3 polyclonal antibody (#ZS-7148), avidin—biotin complex (ABC) staining kit, and 3, 3ʹ- diaminobenzidine (DAB) kit for immunohistochemistry were purchased from Beijing Zhongshan Golden bridge Biotechnology Co, Ltd(Beijing; China). Microtome and microscope named Leica1900 and an Olympus BX51 were also used in this study.

### 4: Establishment of the ovariectomized rat model

20 rats were randomly divided into four groups, and each group consisted of 5 rats: the sham-operated group (SHAM), the ovariectomized group (OVX), the OVX+ E group (OVX group treated with estradiol valerate), and the OVX+ iCR group (OVX group treated with the isopropanolic extract of *Cimicifuga racemosa*). The SHAM group and OVX group used distilled water to instead the drugs. The surgery procedures were as follows: First, the rats were anesthetized, then an incision was made at the midline of the abdomen and the bilateral ovaries were revealed. In the SHAM group, the ovaries were not resected, and the same quantity of fat around the ovaries was removed instead. In the other groups, their bilateral ovaries were ligation and cut off. In the end the abdominal cavity was closed. The rats were given 2 weeks for wound healing after the operation.

### 5: Medication

Both of the estradiol valerate(Bujiale; 1mg per tablet) and *Cimicifuga racemosa* (Remifemin tablets; 20mg crude drug per tablet), used in this study, are commercially available. The manufacturers of the two drugs were Bayer Health Care Co, Ltd (batch number 256A 2; Guangzhou, China) and Schaper & Brümmer Ltd & Co KG (batch number 123813; Germany).

In order to form a uniform liquid, two types of tablet were dissolved in distilled water respectively and the ultrasonic treatment was used. The concentration of the estradiol was 0.2 mg/ml and the iCR was 12mg/ml. 2 weeks after the operation, the rats underwent daily gavage from 8:30 to 9:30 a.m. for 4 weeks. The doses of medication were as follows: SHAM group, 10 ml/kg distilled water; OVX group, 10 ml/kg distilled water; OVX+ E group, 0.8 mg/kg estradiol [21]; OVX+ iCR group, 60 mg/kg *Cimicifuga racemosa* (crude drug). All rats were weighed every three days and the dosage was readjusted according to the variation in body weight.

### 6: Section preparation and H&E staining

The lacrimal and submandibular glands were excised, weighed, and placed in 4% buffered paraformaldehyde (pH 7.4) for 24 h. The fixed tissues were processed routinely and embedded in paraffin for histological and immunohistochemical examination. Paraffin sections (thickness 5 μm) were stained with hematoxylin and eosin (H&E).

### 7: Transmission electron microscopy

The lacrimal and submandibular glands were cut into small pieces (1 mm^3^) and immersed in 2% buffered paraformaldehyde 1.25% glutaraldehyde (pH 7.4) for 2 h. Ultrathin sections were double stained with 4% uranyl acetate and 0.1% lead citrate and examined with a JEM-2100 electron microscope (JEOL, Japan). Each image was obtained under the same conditions, such as brightness and contrast, for a better comparison of density among different groups.

### 8: Immunohistochemical staining

Immunohistochemical staining was then performed. First, Paraffin sections were dewaxed, hydrated, and then incubated in 3%H2O2 for 20 min to block endogenous peroxidase. Second, placed the sections in goat serum for 2 h, and then incubated in rabbit anti-rat Cu-Zn SOD multiclonal antibody (1:500 dilution; Santa Cruz) and rabbit anti-rat Casp-3 multiclonal antibody (1:100 dilution; Beijing Zhongshan Golden bridge Biotechnology Co, Ltd) at 4°C for 24 h. Then the biotinylated goat anti-rabbit IgG serum and ABC solution (Beijing Zhongshan Golden bridge Biotechnology Co, Ltd) were superposed the sections successively, and each reagent was allowed to react at room temperature for 3 h. In the end, the sections were stained with DAB kit, and the immunoreactive products were brown. The negative control were processed in the same manner as described above, however, the rabbit anti-rat Cu-Zn SOD and Casp-3 polyclonal antibody were replaced with 0.1 mol/L PBS.

### 9: Data collection and statistics

All the sections were observed under a light microscope (BX51; Olympus, Japan) and the images were collected using an image analysis system (Quantimet 570c; Leica), with the magnification ×400.

In the H&E sections, an area of at least 100 acinar cells in randomly selected fields were measured, using Image-Pro Plus 6.0 software to analysis the percentage of acinar and duct cells of area in the gland lobule in each vision.

The percentage of Casp-3 positive cells: Each immunohistochemical film randomly selected five fields of vision. First of all, the number of positive acinar cells in each photo was counted as “a”, and then the total number of the acinar cells in the image was counted as “b”, “a/b” giving the percentage of positive cells in each film, and finally the average of the five percentages was taken, to obtain the ratio of positive cells of each rat.

The average optical density of Casp-3 and Cu-Zn SOD: Five fields of vision were selected randomly in each immunohistochemical film. Every photograph was obtained under the same conditions of brightness and contrast ratio. The Image-Pro Plus 6.0 software was used to determine the average optical density analysis. The area for measurement was circled manually (area of interest, AOI), and different structures were analyzed respectively, including the acinar cells, glandular epithelial cells, striated ducts and granular convoluted tubule (GCT)). Next, the area of the AOI (Area) and the integral optical density (IOD) value was measured, and the IOD/Area was calculated to obtain of the mean IOD in each image. Finally, the average of the mean IOD in each image was used to determine the final mean IOD of each rat.

The data were calculated as the mean ± standard deviation and analyzed using SPSS17.0, with P value <0.05 indicating statistical significance. Because the levels of variance in the percentage of acinar and duct cells area in the lobule of lacrimal gland were heterogeneous using Levene test, the Kruskal-Wallis H test and Mann-Whitney U tests were used to analyze these data. Other data was processed using one-way analysis of variance (one-way ANOVA), followed by LSD post hoc test.

## Results

### 1: The basic condition of the animals

The body weight (BW) and the body weight gain (BWG) of the OVX group increased significantly (p<0.01 vs. SHAM group). Estradiol therapy was shown to significantly suppress body weight (p<0.01 vs. OVX group), and no difference was observed compared with the SHAM group (p>0.05 vs. SHAM group). However, in the OVX + iCR group, both of the BW and BWG was not significantly suppressed (p<0.01 vs. SHAM group). The weight of uterus was significantly reduced in both the OVX and OVX+ iCR group (p<0.01 vs. SHAM group), but not in the OVX+ E group (p>0.05 vs. SHAM group). In the OVX group, the weight of lacrimal gland (LGW) increased significantly (p<0.05 vs. SHAM group), but no difference in LGW/BW (p>0.05 vs. SHAM group). The iCR treatment resulted in a greater SGW/BW value than that of the OVX group (p<0.05 vs. OVX group). No statistical difference was found between the SHAM, OVX and OVX+E groups (p>0.05) (Table 1).

**Table 1:** Effects of ovariectomy, estrogen and iCR therapy on body weight, lacrimal gland and submandibular gland weight.

	SHAM	OVX	OVX+E	OVX+iCR
N	5	5	5	5
**BW initial(g)**	251.80±6.22	258.60±6.58	254.80±17.21	265.20±5.26
**BW final(g)**	278.60±9.63^d^ ^g^	346.40±30.63^b^ ^e^	297.00±32.45^d^ ^g^	340.40±19.14^b^ ^e^
**WGR(%)**	10.67±3.96^d^ ^g^	34.03±12.63^b^ ^e^	16.65±10.65^d^	28.32±5.97^b^
**UW(mg)**	400.08±13.76^d^ ^g^	88.82±4.35^b^ ^e^	400.24±9.98^d^ ^g^	86.44±2.32^b^ ^e^
**LGW(mg)**	124.37±11.67^c^	158.84±0.019^a^	145.16±17.55	139.58±25.94
**LGW/BW final (mg/g)**	0.45±0.053	0.46±0.07	0.49±0.032	0.47±0.05
**SW(mg)**	269.28±27.05	288.06±40.43	288.30±26.06	300.66±18.87
**SW/BW final (mg/g)**	0.96±0.10	0.83±0.13^f^	0.98±0.13	1.02±0.09^c^

BW, body weight; WGR, weight gain rate; UW, uterus weight; LGW, lacrimal gland weight; LGW/BW final, lacrimal gland and final body weight ratio; SW, submandibular gland weight; SW/BW final, submandibular gland and final body weight ratio. Data are expressed as mean± SEM.

a means p<0.05 vs. SHAM;

b means p<0.01 vs. SHAM;

c means p<0.05 vs. OVX;

d means p<0.01 vs. OVX;

e means p<0.01 vs. OVX+E;

f means p<0.05 vs. OVX+ iCR;

g means p<0.01 vs. OVX+ iCR.

### 2: H&E staining

**Fig 1:**
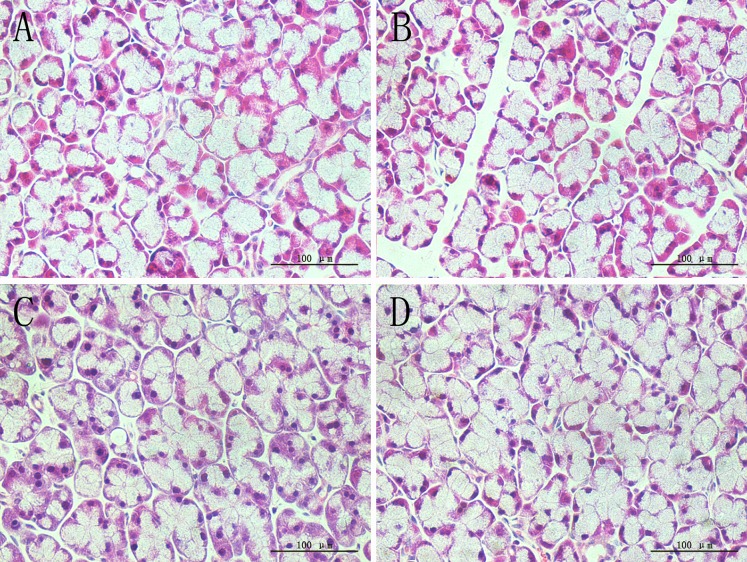
H&E staining of lacrimal gland. A, SHAM; B, OVX; C, OVX + E; D, OVX + iCR. All scale bars represent 100 μm (magnification ×400). Compared with the SHAM group, the OVX group showed a sparse acinar cells, and the intercellular space was increased, suggested that cell atrophy was present, while estradiol valerate and iCR treatment can protected the glands from ovariectomy-induced cell atrophy to a certain extent.

**Fig 2:**
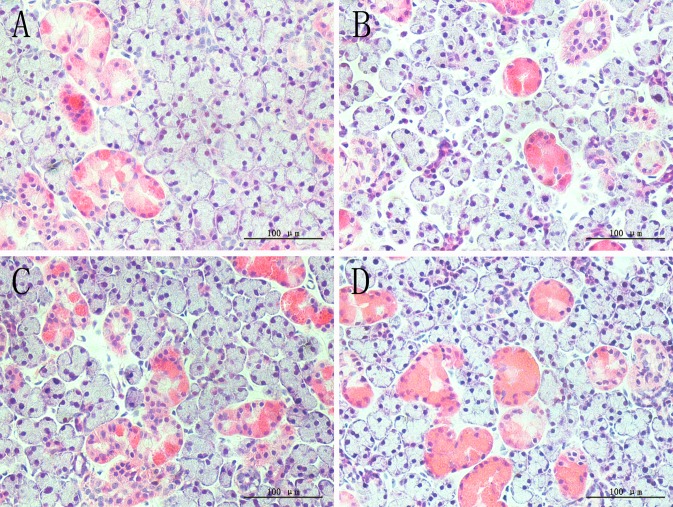
H&E staining of submandibular gland. A, SHAM; B, OVX; C, OVX + E; D, OVX + iCR. All scale bars represent 100 μm (magnification ×400). Coincidence with the lacrimal gland, sparse acinar cells and increased intercellular space was commonly seen in the OVX group, while both of the treatment can improve the condition of ovariectomy-induced lesions.

In both glands, the OVX group showed sparse acinar cells, and the intercellular space was increased, this may suggested that cell apoptosis and atrophy were present, while estradiol valerate and iCR treatments significantly protected glands from ovariectomy-induced lesions (Fig. 1 and Fig. 2). The percentage of acinar and duct cells area in gland lobule indicated the severity of cell contraction.

#### 2.1: Lacrimal gland

**Fig 3:**
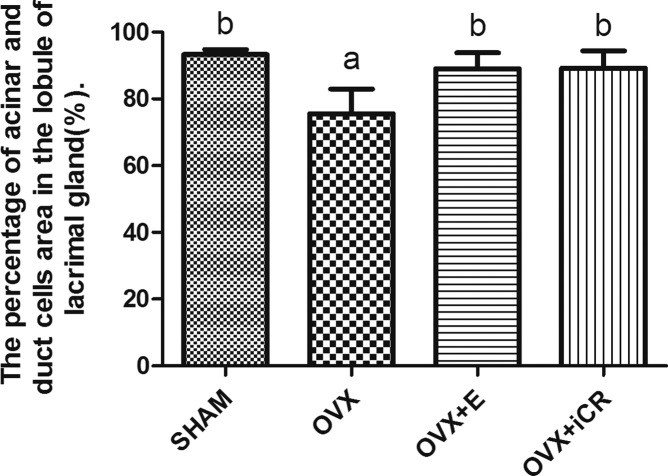
The percentage of acinar and duct cells area in the lobule of lacrimal gland. a means p<0.01 vs. SHAM; b means p<0.01 vs. OVX.

The percentage of acinar and duct cells of the gland lobule was decreased significantly (p<0.01 vs. SHAM group). After the treatment of estradiol valerate and iCR, it increased significantly (p<0.01 vs. OVX group). No significant difference was found among the SHAM, OVX+ E and OVX+ iCR groups (p>0.05) (Fig. 3).

#### 2.2: Submandibular gland

**Fig 4:**
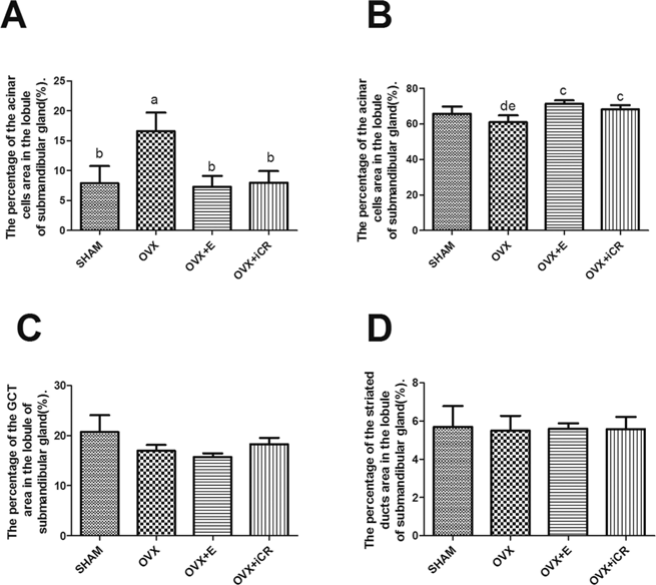
The percentage of acinar and duct cells area in the lobule of submandibular gland. A, The percentage of intercellular space area in the lobule of submandibular gland. B, The percentage of acinar cells area in the lobule of submandibular gland. C, The percentage of GCT area in the lobule of submandibular gland. D, The percentage of striated ducts area in the lobule of submandibular gland. a means p<0.01 vs. SHAM; b means p<0.01 vs. OVX; c means p<0.05 vs. OVX; d means p<0.05 vs. OVX+E; e means p<0.05 vs. OVX+ iCR.

Unlike the lacrimal gland, the structures in the submandibular gland were diverse, which may impact the results. So we used the intercellular space to stand for the contraction of cells. Compared with the other groups, the percentage of intercellular space area in the lobule of submandibular gland increased markedly (p<0.01) (Fig. 4, A). The percentage of acinar cells area decreased significantly in the OVX group compared with the OVX+ E and OVX+ iCR groups (p<0.05), and there was no difference between the SHAM, OVX+E and OVX+ iCR groups (p>0.05) (Fig. 4, B). No difference was found in the percentage of GCT and striated ducts area in the lobule of submandibular gland (p>0.05) (Fig. 4, C D).

### 3: Transmission electron microscopy

#### 3.1: Lacrimal gland

**Fig 5:**
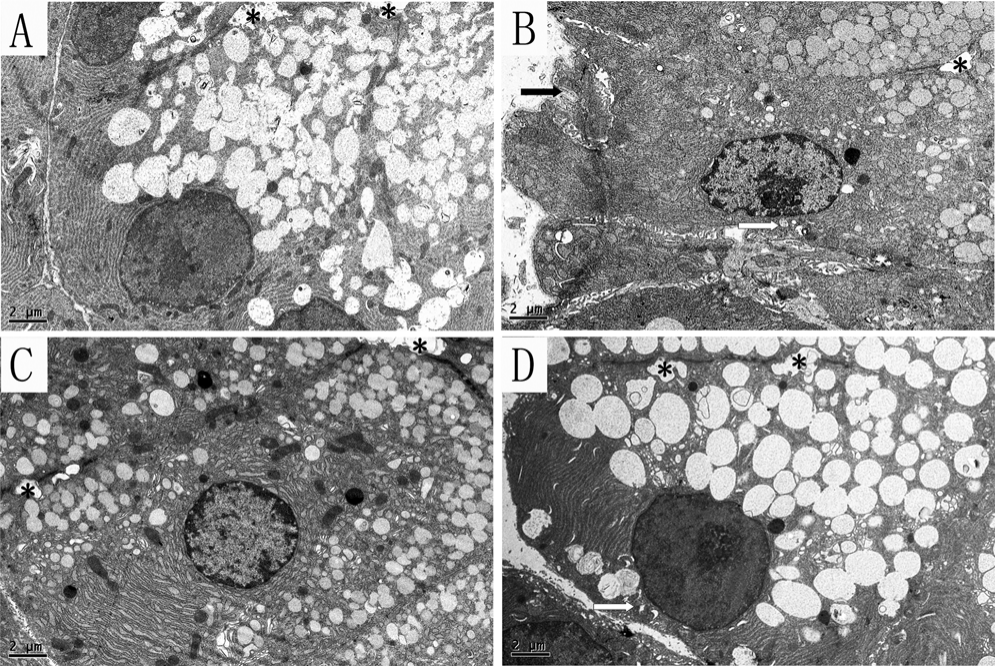
The acinar cells in the lacrimal gland. A, SHAM; B, OVX; C, OVX + E; D, OVX + iCR. All scale bars represent 2 μm. * stand for the lumen. The white arrows indicate the mitochondria lesion, and the black arrow represents the cell membrane sag. Compared with the SHAM group (Fig. 5, A), the endoplasmic reticulum was diluted and arranged in a disorderly fashion in the OVX groups. Most of the mitochondria appeared to be swollen, with dissolution and crest fracture. Moreover, the diameter of the white secretory granules seemed decreased obviously. The cell shrink, with sag on the membrane and an increased intercellular space, was often seen (Fig. 5, B). Estrogen treatment did not ameliorate the enlargement of endoplasmic reticulum, but the mitochondria seemed normal—ridge fracture was rarely seen. Similar to OVX group, the diameter of the secretory granules decreased also, but the extent of cell shrink was more slightly (Fig. 5, C). Interestingly, the iCR therapy was shown to ameliorate the expansion of endoplasmic reticulum, but not the mitochondria lesions. The diameter of the secretory granules was analogous to SHAM group, and the cell atrophy was rarely observed (Fig. 5, D).

Under the electron microscope, we mainly observed acinar cells in the lacrimal gland, which is the primary place where tears are formed. In the SHAM group, the nucleus and organelle were located on the basolateral side, with well-arranged endoplasmic reticulum and healthy mitochondria (Fig. 5, A). Compared with the SHAM group, the endoplasmic reticulum was diluted and arranged in a disorderly fashion in the OVX groups. Most of the mitochondria appeared to be swollen, with dissolution and crest fracture. Moreover, the diameter of the white secretory granules seemed decreased obviously (Fig. 5, B). The shrink cells, with sag on the membrane and an increased intercellular space, were often seen. Estrogen treatment did not ameliorate the enlargement of endoplasmic reticulum, but the mitochondria seemed normal—ridge fracture was rarely seen. Similar to OVX group, the diameter of the secretory granules decreased also, but the extent of shrink cells was more slightly (Fig. 5, C). Interestingly, the iCR therapy was shown to ameliorate the expansion of endoplasmic reticulum, but not the mitochondria lesions. The diameter of the secretory granules was analogous to SHAM group, and the cell atrophy was rarely observed. (Fig. 5, D).

**Fig 6:**
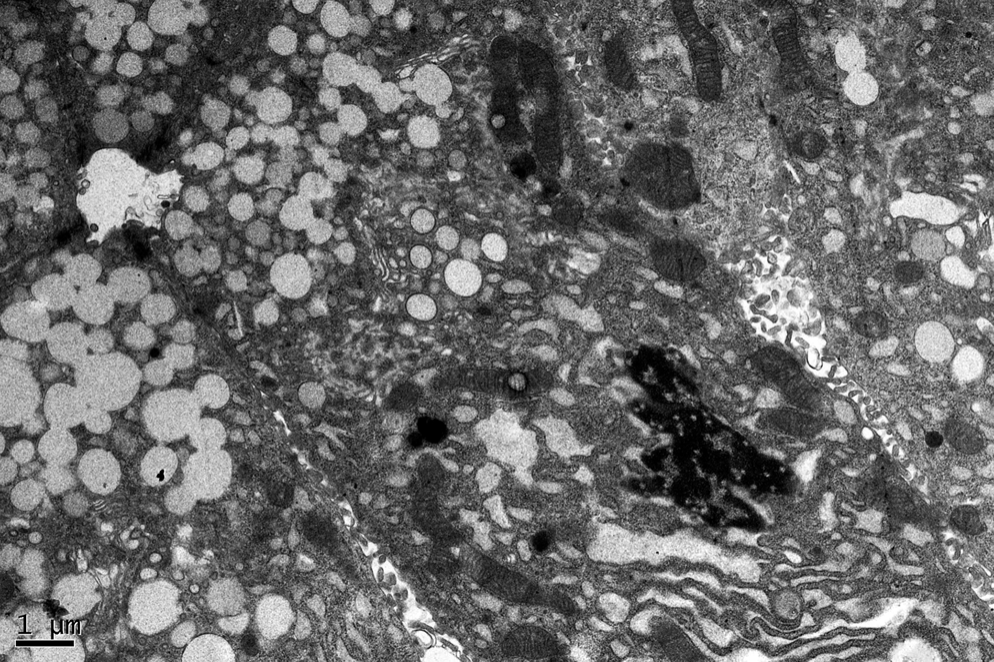
The apoptosis cell of OVX group in lacrimal gland. The apoptosis cells were seen in the OVX group, which had an irregular nuclear with chromatin pyknosis and lost nuclear membrane. Besides, the endoplasmic reticulum was cracked. The scale bar represents 1μm.

Moreover, the apoptosis cells were seen in the OVX group, which had an irregular nuclear with chromatin pyknosis and lost nuclear membrane. Besides, the endoplasmic reticulum was cracked (Fig. 6).

#### 3.2: Submandibular gland

**Fig 7:**
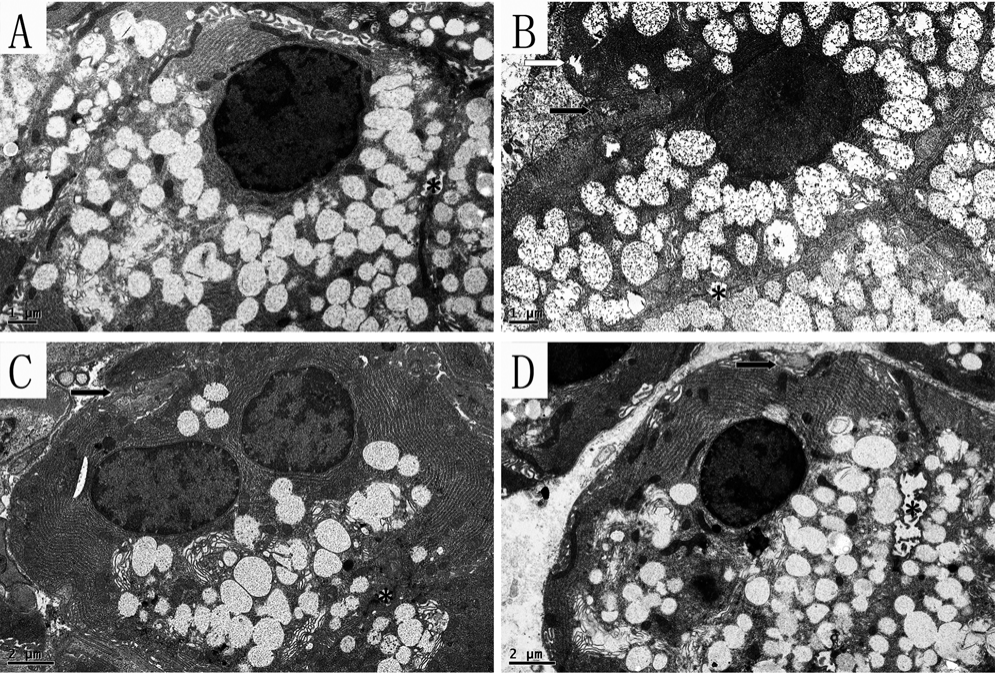
The acinar cells in the submandibular gland. A, SHAM; B, OVX; C, OVX + E; D, OVX + iCR. The scale bars in A, B represent 1μm, and in C, D represent 2 μm. The white arrow indicates the cell membrane sag, and the black arrows represent the cell membrane sag. * stand for the lumen. Compared with the SHAM group, obvious swelling occurred in the mitochondria of the OVX group with dissolution and crest fracture, but unlike the lacrimal gland, there was no obvious expansion of the endoplasmic reticulum. Moreover, the cells showed obvious atrophy with membrane sag. In the OVX + E and OVX + iCR groups, the number of mitochondrial lesions decreased, but the number of secretory granules seemed to decrease compared with the SHAM and OVX groups. The indentation of the cell membrane was improved by the two drugs.

Acinar cells, GCT and striated ducts were observed in submandibular gland. Amylase, mucin and other main ingredients of saliva are synthesized in the acinar cells, and then secret into the lumen to form saliva. Many secretory granules are located near the lumen side, while the cell nucleus, mitochondria and endoplasmic reticulum are generally located on the basal side (Fig. 7, A). Compared with the SHAM group, obvious swelling occurred in the mitochondria of the OVX group with dissolution and crest fracture, but unlike in the lacrimal gland, there was no obvious expansion of the endoplasmic reticulum. Moreover, the cells showed obvious atrophy with membrane sag (Fig. 7, B). In the OVX+ E and OVX+ iCR groups, the number of mitochondrial lesions decreased. However, the indentation of the cell membrane was improved by the two drugs, suggesting that both drugs had effect on ameliorating cell atrophy which was coincidence with the result of H&E staining, but the number of secretory granules seemed to decrease compared with the SHAM and OVX groups (Fig. 7, C D).

**Fig 8:**
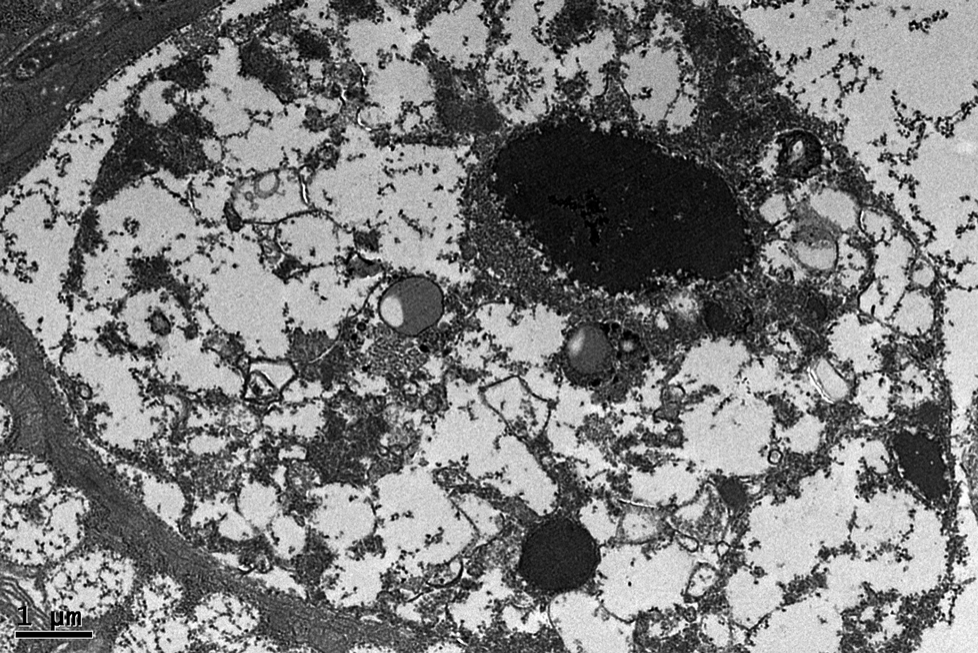
The apoptosis cell of OVX group in submandibular gland. The apoptosis cells were commonly seen in OVX group, with a rupture of membrane structures, including the cell membrane, mitochondria membrane and nuclear membrane, etc. The scale bar represents 1μm.

In addition,the apoptosis cells were commonly seen in OVX group, with a rupture of membrane structures, including the cell membrane, mitochondria membrane and nuclear membrane, etc (Fig. 8).

**Fig 9:**
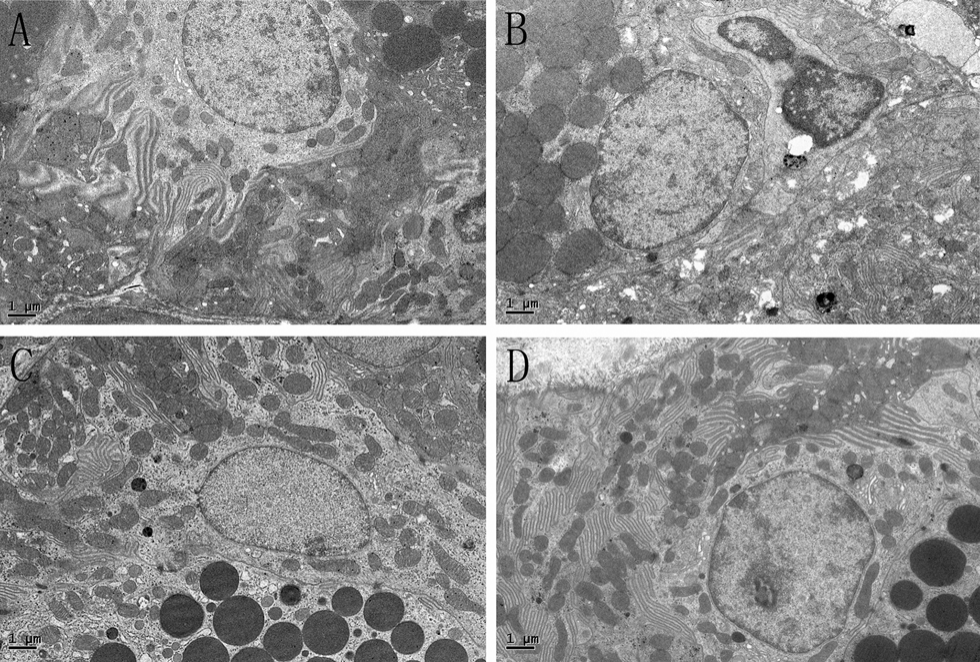
The GCT in the submandibular gland. A, SHAM; B, OVX; C, OVX + E; D, OVX + iCR. Scale bars represent 1 μm. In the SHAM group, the cell plasma was filled with a high density of secretory granules, and the nucleus was located on the basolateral side of the cell with normal chromatin morphology, and mitochondria were intact. In the OVX group, many of the mitochondria were swollen, with double membrane structure damage and crest fracture, and “balloon-like” mitochondria were common. The condensed chromatin morphology was inside the cells, probably resulted- from the mutual ingestion of apoptotic cells. The color density of the secretory granules was reduced, with a gray appearance. In the OVX+ E and OVX+ iCR groups, most of the mitochondrial structure was normal, the distribution of chromatin was uniform, and the density of black secretory granules was similar to that of the SHAM group.

The granular convoluted tubules are the serous salivary epithelial cells for rodents, similar to human serous acinar cells. These cells synthesize and excrete neutral mucopolysaccharides and a variety of biologically active polypeptides, including amylase [22, 23]. In the SHAM group, the cell plasma was filled with a high density of secretory granules, and the nucleus was located on the basolateral side of the cell with normal chromatin morphology, and mitochondria were intact (Fig. 9, A). In the OVX group, many of the mitochondria were swollen, with double membrane structure damage and crest fracture, and “balloon-like” mitochondria were common. There was condensed chromatin morphology inside the cells, probably resulting from the mutual ingestion of apoptotic cells (Fig. 9, B). The color density of the secretory granules was reduced, with a gray appearance. In the OVX+ E and OVX + iCR groups, most of the mitochondrial structure was normal, the distribution of chromatin was uniform, and the density of black secretory granules was similar to that of the SHAM group (Fig. 9, C D).

**Fig 10:**
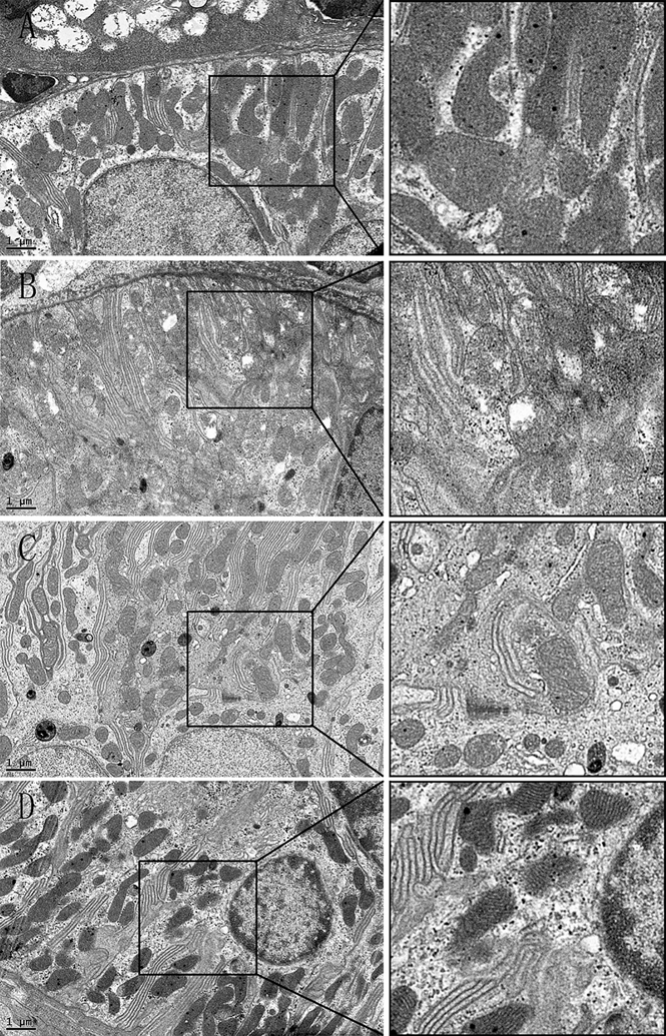
The striated duct of the submandibular gland. A, SHAM; B, OVX; C, OVX+ E; D, OVX+ iCR. Scale bars represent 1 μm. In the SHAM group, there was no obvious swelling or crest fracture in the mitochondria of the striated ducts. In contrast, many of the mitochondria were swollen, with double membrane structure damage, crest fracture in OVX group, but the condensed chromatin morphology was rarely observed. Both treatments improved this deleterious effect, resulting in mitochondria of normal appearance.

The principal function of striated ducts is to absorb Na+ and Cl—ions from the primary saliva, and secrete K+ and HCO3—ions [22]. Cell infolding is present on the basal side of a striated duct, with many mitochondria among the folds, in order to provide energy for the active transport of ions. In the SHAM group, there was no obvious swelling or crest fracture in the mitochondria of the striated ducts (Fig. 10, A). In contrast, many of the mitochondria were swollen, with double membrane structure damage, and crest fracture in OVX group, but condensed chromatin morphology was rarely observed (Fig. 10, B). Both treatments improved this deleterious effect, resulting in mitochondria of normal appearance (Fig. 10, C D).

### 4: Casp-3 expression

**Fig 11:**
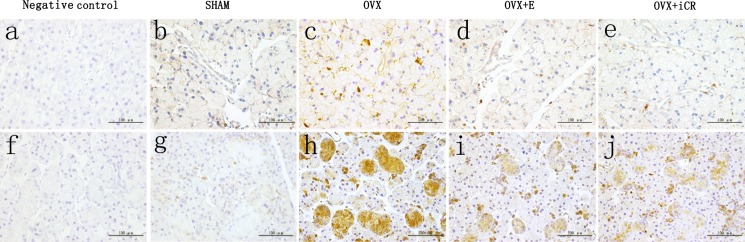
Casp-3 expression. a-e, lacrimal gland; f-j, salivary gland. Scale bars represent 100 μm (magnification ×400). The positive staining was brown, and there was no brown color in the negative control. In the lacrimal gland, the positive staining was expressed mainly in the acinar cells. In the submandibular gland, Casp-3 was mainly expressed in the acinar cells and the cytoplasm of GCT and striated duct epithelial cells.

**Fig 12:**
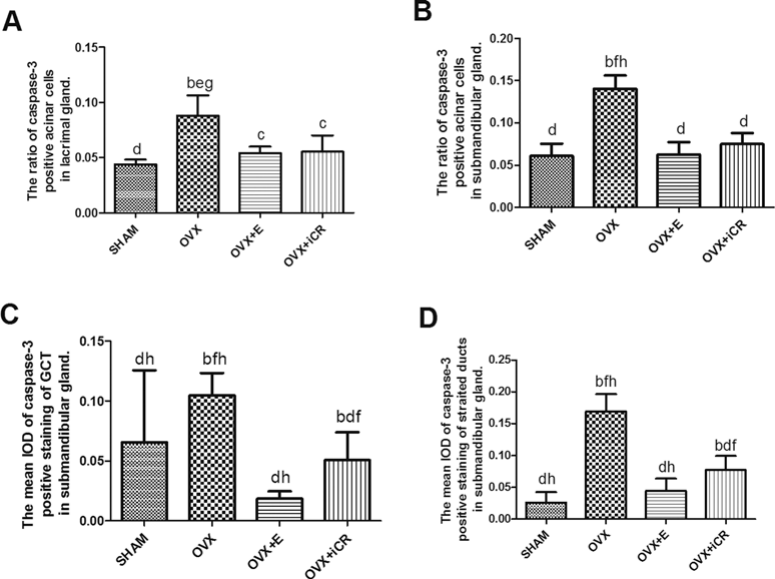
The expression intensity of caspase-3 in the two kinds of gland. A: The ratio of casp-3 positive acinar cells in lacrimal gland. B: The ratio of casp-3 positive acinar cells in submandibular gland. C: The IOD of casp-3 positive staining in GCT of the submandibular gland. D: The IOD of casp-3 positive staining in the striated ducts of the submandibular gland. a means p<0.05 vs. SHAM; b means p<0.01 vs. SHAM; c means p<0.05 vs. OVX; d means p<0.01 vs. OVX; e means p<0.05 vs. OVX+ E; f means p<0.01 vs. OVX+ E; g means p<0.05 vs. OVX+ iCR; h means p<0.01 vs. OVX+ iCR.

The positive staining was brown, and there was no brown color in the negative control. In the lacrimal gland, the positive staining was expressed mainly in the acinar cells (Fig. 11, a-e). The ratio of Casp-3 positive acinar cells in the OVX group increased significantly (p < 0.01 vs. SHAM group). The estradiol and iCR treatments suppressed the Casp-3 expression significantly (p < 0.05 vs. OVX group). No significant difference was found among the SHAM, OVX+ E and OVX+ iCR groups (p > 0.05) (Fig. 12, A).

In the submandibular gland, Casp-3 was mainly expressed in the acinar cells and the cytoplasm of granular curved tubules and striated duct epithelial cells. In contrast to the SHAM group, the ratio of Casp-3 positive cells increased significantly in the OVX group (p < 0.01). After treatment with estradiol and iCR, the ratio of positive acinar cells decreased significantly (p < 0.01 vs. OVX group). There was no obvious difference between the OVX+ E and OVX+ iCR groups (p > 0.05) (Fig. 12, B). The mean IOD in the GCT and striated ducts was similar with the result above. The mean IOD increased significantly in the OVX group (p < 0.01 vs. SHAM group), but decreased significantly in the OVX+ E and OVX+ iCR groups (p < 0.01 vs. OVX group). There was no obvious difference in the OVX+ E and SHAM group (p > 0.05). However, in two types of duct, iCR treatment have a higher mean IOD value than OVX+ E and SHAM group (p<0.01 vs. SHAM group) (Fig. 12, C D).

### 5: Cu-Zn SOD expression

**Fig 13:**
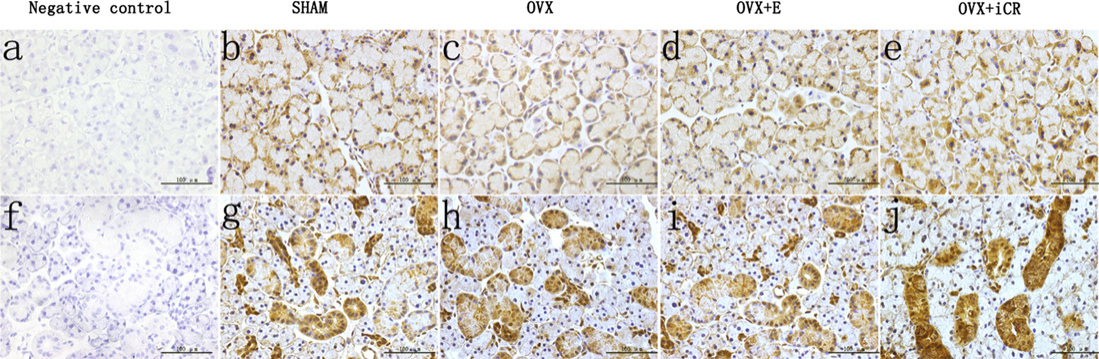
Cu-Zn SOD expression. a-e, lacrimal gland; f-j, submandibular gland. Scale bars represent 100 μm (magnification ×400). In the negative control, no brown precipitation was observed, representing no positive response. In the lacrimal gland, positive staining of Cu-Zn SOD mainly occurred on the basolateral side of the acinar cells, while in the submandibular gland, strong staining was found in the GCT and striated ducts.

In the lacrimal gland, positive staining of Cu-Zn SOD mainly occurred on the basolateral side of the acinar cells, while in the submandibular gland, strong staining was found in the GCT and striated ducts. In the negative control, no brown precipitation was observed, representing no positive response (Fig. 13, a-e).

**Fig 14:**
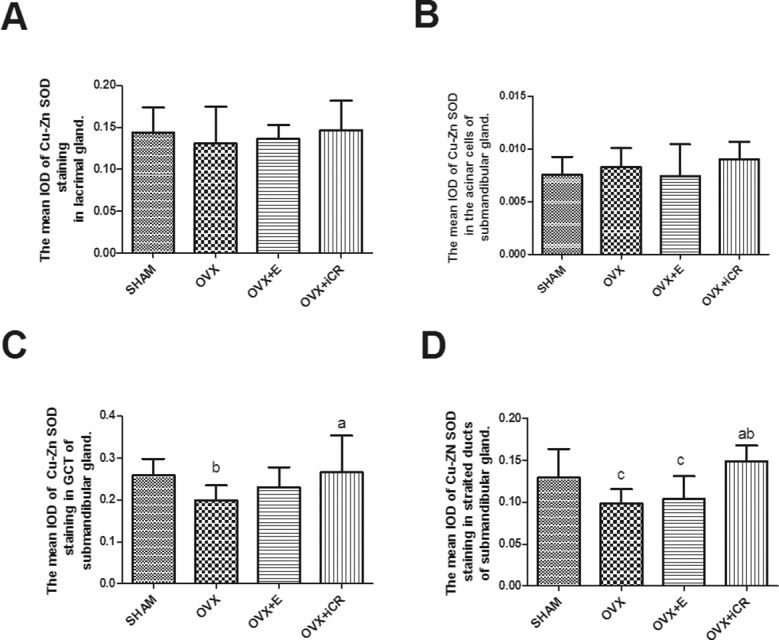
The expression intensity of Cu-Zn SOD in lacrimal and submandibular glands. A: The mean IOD of Cu-Zn SOD staining in lacrimal gland. B: The mean IOD of Cu-Zn SOD staining in the acinar cells of the submandibular gland. C: The mean IOD of Cu-Zn SOD staining in the GCT of the submandibular gland. D: The mean IOD of Cu-Zn SOD staining in the striated ducts of the submandibular gland. a means p<0.05 vs. OVX; b means p<0.05 vs. OVX+ iCR.

In the lacrimal gland, there was no significant difference between the four groups (p > 0.05) in terms of the mean IOD of the acinar and duct cells (Fig. 14, A). However, the number of lachrymal ducts present was insufficient for statistical analysis. In the submandibular gland, the mean IOD of the acinar cells had no significant difference among the SHAM, OVX, OVX + E and OVX + iCR groups (p > 0.05) (Fig. 14, B). Nonetheless, iCR treatment led to a significant increase in the mean IOD of Cu-Zn SOD in the GCT and striated ducts (p < 0.05 vs. OVX group) (Fig. 14, C D).

## Discussion

The present study showed that when the bilateral ovaries were removed, the body weight significantly increased, which was suppressed after treatment with estradiol. These results concurred with those of many earlier studies [24, 25]. Winterhoff et al. found that, with two different dosages of *Cimicifuga racemosa* (50 mg/kg and 100 mg/kg), only the higher dosage led to a down regulation in body weight [26]. This may provide a reasonable explanation as to why iCR had no obvious effect on body weight, as the dosage we used (60 mg/kg) was close to the lower figure. Although we did not measure the level of estrogen in the blood, the uterus weight reflected the estrogen level, which was significantly reduced in the OVX group and OVX+ iCR group. Though same as edocrine gland on maxillofacial, lacrimal and submandibular glands responded differently to ovariectomy and to both types of therapy. The data showed that, compared with the SHAM group, the weight of lacrimal gland increased, but there was no difference in LGW/BW in the OVX group. This phenomenon suggests that growth may accompany an increase in body weight in order to meet functional requirements. However, no difference was observed in submandibular gland weight in the SHAM, OVX and OVX+ E groups. Notably, the OVX+ iCR group showed a remarkable growth in submandibular gland and final body weight ratio compared with the OVX group, suggesting that it can promote gland growth.

According to the two-stage hypothesis proposed by Thaysen, the hyperosmotic or isotonic plasma-like primary saliva is initially secreted by salivary acinar cells [27]. Subsequently, as primary saliva travels through the duct system, salivary ducts reabsorb some electrolytes, modifying the electrolyte composition of the primary saliva. Throughout this process, both the acinar and ductal cells play a vital role and the lesions of the two structures may contribute to a dry mouth sensation. This study found that ovariectomy can induce organelle lesions, cell atrophy and apoptosis, and up regulate Casp-3 expression. Estradiol and iCR therapy can relieve these morphological changes partly and down-regulate the expression of Casp-3. Moreover, iCR can improve the expression of Cu-Zn SOD in the duct cells of submandibular gland.

With electron microscopy, the two therapies revealed different effects—mainly performance related on the ultra-structural appearance of the two types of gland. These effects included the following: 1) expansion of the endoplasmic reticulum; 2) damage to mitochondrial integrity; 3) cell atrophy; 4) an increased level of apoptosis.

The major physiological function of endoplasmic reticulum is protein synthesis. Immune colloidal gold labeling inspection of pituitary cell revealed that the rough endoplasmic reticulum lumen can detect the expression of ER alpha and ER beta [28]. It has been proved that endoplasmic reticulum stress can inhibit the activation Akt pathway, which, in turn, induces the expression of Casp-3, while a low concentration of estrogen (10^–9M^) can counteract endoplasmic reticulum stress and cause the deactivation of Akt, thus inhibiting the expression of Casp-3, resulting in an eventual anti-apoptotic effect [29]. Interestingly, in the lacrimal gland, iCR can significantly improve the expansion of endoplasmic reticulum, while there is no significant improvement in expansion of endoplasmic reticulum with estrogen, indicating that iCR has a different mechanism of action from estrogen. It is speculated that the endoplasmic reticulum of acinar cells in the lacrimal gland do not contain estrogen receptors, and that the estrogen receptors in the cytoplasm mainly exist in mitochondria. Therefore, estrogen therapy has no effect on endoplasmic reticulum. At present, none of the literature has reported that black cohosh can inhibit the expansion of endoplasmic reticulum, so it may be directly related to the anti-apoptotic effect of iCR.

The most important function of mitochondria is to convert chemical energy to ATP, which is involved in many important cell activities. Furthermore, it plays a key role in programmed cell death. A study found that there was existed a functionally active mitochondrial pool of p38β of myocardial cell mitochondria, which can phosphorylate Mn SOD, a critical component of the mitochondrial antioxidant system, in an estrogen-dependent manner [30]. This provides a novel mechanism by which estrogen reduces oxidative stress and protects cardiomyocytes. Thus, according to electron microscopy observations of mitochondria, this study hypothesized that estrogen may play the same role in lacrimal and salivary glands. Lee et al. found that pretreatment with deoxyactein, an extract of iCR, prior to antimycin A exposure, reduced antimycin A-induced cell damage by preventing mitochondrial membrane potential dissipation, complex IV inactivation, ATP loss, [Ca2+] elevation and oxidative stress. Moreover, deoxyactein increased the activation of PI3K (phosphoinositide 3-kinase), Akt (protein kinase B) and CREB (cAMP-response element-binding protein) inhibited by antimycin A [31]. Therefore, the protective effect of iCR on mitochondria may be induced by, for example, deoxyactein.

Atrophy usually results from deterioration in cell function, nutrient supply, or nerve and/or endocrine stimulation. In this experiment, in addition to the SHAM group, the atrophy acinar cells in OVX group were common with sunken cell membranes, and broadening gap, and estrogen and iCR therapy significantly improved the shrinkage phenomenon. This is coincidence with the previous research [32]. Although the iCR treatment cannot react with estrogen receptor, and increase estrogen-dependent gene expression, the protective effects on osteoporosis and alleviation of hot flashes are like estrogen [33]. If cell atrophy is purely due to lack of hormones, then the improvement of cell atrophy with iCR can be attributed to it having estrogen-like effect. Moreover, the estrogen therapy seems not increased the diameter of secretory granules caused by ovariectomy. At present, it is controversial about the effect of estrogen treatment of dry eyes. Song X et al. pointed out that the estrogen play a pathogenetic role in ovariectomy-induced dry eyes[34]. But we can’t conclude the function of secretion was affected, for that the effects of the two drugs on the secretory function were not examined in the study.

Since the discovery that activated Casp-3 changes apoptotic cell structure and causes DNA splitting and cell atrophy, it has been broadly recognized that Casp-3 is a histochemical marker specific for cell apoptosis [35, 36]. Up to now, the function of estrogen on protecting cell apoptosis of lacrimal gland has been opposite. Early research found that estrogen treatment had no effect on lacrimal gland [37]. However, recent research conducted by Azzarolo et al. revealed that 17β-estradiol exhibits remarkable effects such as inhibiting tear gland cell apoptosis, and attenuating lymphocyte infiltration of ovariectomized rabbit [38]. Moreover, both estrogen and androgen have been shown to significantly increase the lipocalin content of tears [39]. Obviously, our study supports the latter observation. It has been well established that estrogen withdrawal can prevent cell death within the salivary gland. Yet, when Tsinti et al. used salivary gland cell culture in vitro, they found that lack of estrogen has no influence on cell apoptosis [40]. This finding may have arisen because the environment in vivo is more complicated than that in vitro. Our study showed that estrogen could inhibit cell apoptosis of submandibular gland. Many studies have indicated that the anti-apoptotic function of estrogen may be attributed to non-genomic mechanisms, mainly through the activation of multiple kinases such as MAPK (ERK1/2 pathway, JNK pathway) and PI3K (PI3K/Akt pathway). Further studies are needed to determine which pathway contributes most in terms of the anti-apoptotic function of estrogen.

The free radical is a highly unstable oxidant, which can cause many diseases, such as inflammation, atherosclerosis and aging, by consuming other molecular electronics, leading to oxidative damage. Cu-Zn SOD, also known as SOD-1, can catalyze the ultra oxygen anion (O2-) and convert it to hydrogen peroxide, and is found in the cytoplasm of free radical scavengers. Its genetic mutations can cause familial amyotrophic lateral sclerosis [41]. It is well known that sex hormone is a natural antioxidant, especially estriol and estradiol. During menstruation, the serum estrogen level is proportional to the level of anti-oxidase and anti-oxidative activity, yet has an inverse correlation with serum lipid peroxide (an oxidative stress production) [42, 43]. These findings indicate that lack of estrogen may be responsible for the reduction in anti-oxidase and the increasement in ROS, and the structural and functional changes that occur after menopause may have a close relationship with the oxidative stress caused by estrogen withdrawal. Kusunoki et al. reported that Cu-Zn SOD positive expression rate in the parotid gland in menopausal rats is lower than that in premenopausal rat, while estrogen treatment can promote expression in postmenopausal rat [19]. Our own study used lacrimal gland and submandibular gland as the experiment material, and showed that there were no remarkable differences in Cu-Zn SOD expression between the OVX and OVX+E groups. This might have been due to the different observation times. Another study, which focused on rat VSMC (vascular smooth muscle cells), showed that 17β-estradiol had no effect on Cu-Zn SOD expression [44]. This might indicate that the level of Cu-Zn SOD expression differs among different tissues. The author infers that the specific distribution of ER in glands contributes to these findings. Researchers have found ER mRNA present in both lacrimal gland and salivary gland, and ERβ is prominent, rather than ERα [45, 46, 47]. Baltgalvis et al. reported that ERα, binding with estrogen, could elevate the expression of SOD and GSH-Px through the activation of MAPK and NF-κB in skeleton muscle cell [48]. These findings revealed to us that Cu-Zn SOD expression may be mediated by ERα. While in these glands, a predominance of ERβ may determine that estrogen has little effect on the level of expression.

However, iCR treatment can significantly increase the expression of Cu-Zn SOD in the duct cells of submandibular gland. Cu-Zn SOD can neutralize ROS in cell plasma and mitochondria, and attenuate damage to the mitochondria of duct cells. Research on the cultured salivary gland cells of rat and human found that estrogen exerts an inhibitory effect on IFNγ-mediated Fas expression[40]. When estrogen is deficient, Fas can transport apoptosis signal through caspase and mitochondrial pathways [49]. Furthermore, in Cu-Zn SOD transgenic rat, the over-expression of the protein as revealed by a study on Alzheimer’s disease, can apparently inhibit Fas expression in rat cerebral cortex and function as a neuron protective factor [50]. We may conclude that iCR can hinder cell death by stimulating Cu-Zn SOD expression, which in turn can inhibit Fas expression. Campos et al. reported that *Cimicifuga racemosa* can significantly increase Cu-Zn SOD activity in liver cells. However, they considered the result to be a response to oxidative stress, especially the elevated H2O2 level in the liver cells, which may have been caused by hepatotoxicity of *Cimicifuga racemosa* [51]. Although the study used Wistar rats that had undergone ovariectomy and unilateral nephrectomy, which is different from the model we used in our study, further investigation is needed to determine whether iCR is protective or toxic.

Moreover, in addition to anti-apoptosis and antioxidant, estrogen and *Cimicifuga racemosa* treatments may influence both glands through other mechanisms. L Deng et al. demonstrated that the estrogen-induced gene EIG121 can regulate autophagy and promotes cell survival under stress [52]. Two new cycloartenol triterpene saponins isolated from *Cimicifuga simplex* could carry out anti-proinflammatory activities by LPS-stimulated IL-6, IL-23 and TNF-a genes expression in RAW cells [53]. Whether these mechanisms also exist in the two glands need further research.

This study has several limitations. The sample size was small—just sufficient for an exploration of the effect of iCR on lacrimal gland and submandibular gland. In future studies, the sample size will be increased to avert individual variation. Furthermore, we failed to compare the effects of the two drugs we investigated on the secretory function, which will be examined in our further study. Last, but not least, it is important to identify the active ingredient in iCR and its protective or harmful effects on glands. This will provide valuable insight for the development of clinical medication.

## Conclusion

Our results indicate that ovariectomy can cause changes to organelles, such as mitochondria crest fracture and endoplasmic reticulum enlargement. Moreover, cell atrophy and apoptosis are common in both types of gland. It is interesting to note that, in the acinar cells of lacrimal gland, the performance of the two kinds of drug treatment differs: black cohosh can significantly improve the endoplasmic reticulum expansion in the lacrimal gland, and estrogen has a more significant protective effect on the mitochondria. Both treatments can reduce the up-regulated expression of Casp-3 induced by ovariectomy, and black cohosh can significantly increase Cu-Zn SOD expression in the duct cells of the submandibular gland. This was the first study to investigate the effects of *Cimicifuga racemosa* on lacrimal and submandibular glands, and revealed that there are many similarities and differences in the effects of both therapies. Such research will inform the experimental study and clinical application of black cohosh.

## Supporting Information

S1 Fig The percentage of acinar and duct cells area in the lobule of lacrimal gland. (XLSX)

S2 Fig The percentage of acinar and duct cells area in the lobule of submandibular gland. (XLSX)

S3 Fig The expression intensity of caspase-3 in the two kinds of gland. (XLSX)

S4 Fig The expression intensity of Cu-Zn SOD in lacrimal and submandibular glands. (XLSX)

S1 Table Effects of ovariectomy, estrogen and iCR therapy on body weight, lacrimal gland and submandibular gland weight. (XLSX)
